# Supplementary material for: Mass homicide by firearm in Canada: Effects of legislation
Source: PLoS One. 2023 Feb 3;18(2):e0266579. doi: 10.1371/journal.pone.0266579 (PMC9897543; doi:10.1371/journal.pone.0266579)
Supplement: S1 Appendix — Table 1A reports sensitivity testing a year after interventions to account for delayed implementation. Table 1B reports findings of sensitivity testing of the model. (DOCX) [file pone.0266579.s001.docx]

S1 Appendix. Sensitivity Testing.

Table 1A. Sensitivity Testing a year after interventions to account for delayed implementation.

| **Intervention** | **Level Rate Ratio^1^** | **95% CI^3^** |  | **Trend Rate**  **Ratio^2^** | **95% CI^3^** |
| --- | --- | --- | --- | --- | --- |
|  |  |  |  |  |  |
| **Incidents** |  |  |  |  |  |
| 1981 | 0.45 | 0.13, 1.50 |  | **1.38** | **1.02, 1.87** |
| 1995 | 0.62 | 0.13, 3.11 |  | 1.03 | 0.95, 1.12 |
| 2002 | **1.87** | **1.01, 3.48** |  | 1.00 | 0.97, 1.02 |
|  |  |  |  |  |  |
| **Total Death Rate** |  |  |  |  |  |
| 1981 | 0.64 | 0.23, 1.78 |  | **1.41** | **1.16, 1.70** |
| 1995 | 0.82 | 0.23, 2.93 |  | 0.98 | 0.92, 1.04 |
| 2002 | 1.37 | 0.78, 2.40 |  | 0.99 | 0.96, 1.01 |
|  |  |  |  |  |  |
| **Male Death Rate** |  |  |  |  |  |
| 1981 | 0.53 | 0.20, 1.37 |  | **1.51** | **1.26, 1.80** |
| 1995 | 1.85 | 0.23, 14.86 |  | 1.00 | 0.91, 1.09 |
| 2002 | 3.87 | 1.82, 8.22 |  | 0.98 | 0.95, 1.00 |
|  |  |  |  |  |  |
| **Female Death Rate** |  |  |  |  |  |
| 1981 | 0.39 | 0.06, 2.68 |  | 1.63 | 1.15, 2.32 |
| 1995 | 1.53 | 0.33, 7.12 |  | 0.99 | 0.90, 1.08 |
| 2002 | 0.91 | 0.41, 2.02 |  | 1.00 | 0.97, 1.02 |

^1^ The rate ratio of the level or immediate impact of firearm mortality relative to non-firearm mortality after each year of legislation implementation which is the β_5_ coefficient. A rate ratio greater than 1 suggests that the level of firearm mortality increased greater than the level in non-firearm mortality, while a ratio less than 1 suggests there is a decrease in the level of firearm compared to non-firearm mortality

^2^ The rate ratio of the trend of firearm mortality relative to non-firearm mortality after each year of legislation implementation which is the difference-in-differences regression results. A rate ratio greater than 1 suggests that the trend of firearm mortality increased greater than the trend in non-firearm mortality, while a ratio less than 1 suggests there is a decrease in the trend of firearm compared to non-firearm mortality.

^3^ CI – confidence interval

Table 1B. Sensitivity Testing of Model. Sensitivity tests were performed to determine at what point the model could detect changes in the level (impact) or trend of firearm mass homicide rates or incidences. After each year of interest, 1980, 1995, or 2001 the data was modified as follows: the incidence rates were individually subtracted by an integer, and/or a rolling 0.5 decrease over two years, and deaths were subtracted by an integer and the data processed by the model. The reduction factor was increased sequentially until a significant (p < 0.05) impact was obtained. As well a percent decrease per year in incidence rate or deaths was also performed on the data after each year of interest until a significant trend was obtained. These results shown in the chart below. For example, in 1980 the model would have detected significance if there were 1.5 less events a year or a change in the number of events by 10% year over year, and 5 less deaths a year or a change in the number of deaths by 10% a year.

| **Year** |  | **Incidence** | | **Deaths** | |
| --- | --- | --- | --- | --- | --- |
|  |  | **Impact**  **(events)** | **Trend** | **Impact**  **(deaths)** | **Trend** |
| **1980** |  | 1.5 | 10% | 5 | 10% |
| **1995** |  | 1.5 | 12% | 4 | 4% |
| **2001** |  | 1.5 | 8% | 5 | 5% |
