# Supplementary material for: Mass homicide by firearm in Canada: Effects of legislation
Source: PLoS One. 2023 Feb 3;18(2):e0266579. doi: 10.1371/journal.pone.0266579 (PMC9897543; doi:10.1371/journal.pone.0266579)
Supplement: S1 File — S1 Table reports the number of victims per incident in firearm and non-firearm-related homicides with three or more victims, Canada, 1974 to 2020. S2 Table reports the number of victims per incident in firearm and non-firearm-related homicides by sex, Canada, 1974 to 2020. (DOCX) [file pone.0266579.s002.docx]

Supplementary Table 1

Number of victims per incident in firearm and non-firearm-related homicides with three or more victims, Canada, 1974 to 2020

Supplementary Table 2

Number of victims per incident in firearm and non-firearm-related homicides by sex, Canada, 1974 to 2020
